# The Pathogenesis of Nonalcoholic Fatty Liver Disease: Interplay between Diet, Gut Microbiota, and Genetic Background

**DOI:** 10.1155/2016/2862173

**Published:** 2016-05-09

**Authors:** Jinsheng Yu, Sharon Marsh, Junbo Hu, Wenke Feng, Chaodong Wu

**Affiliations:** ^1^Department of Genetics, Washington University School of Medicine, St. Louis, MO 63110, USA; ^2^Faculty of Pharmacy and Pharmaceutical Sciences, University of Alberta, Edmonton, AB, Canada T6G 2H7; ^3^Department of General Surgery, Tongji Hospital, Huazhong Science & Technology University, Wuhan, Hubei 430030, China; ^4^Department of Medicine, University of Louisville, Louisville, KY 40208, USA; ^5^Department of Nutrition and Food Science, Texas A&M University, Houston, TX 77843, USA

## Abstract

Nonalcoholic fatty liver disease (NAFLD) is the most common chronic liver disease in the world, and it comprises a spectrum of hepatic abnormalities from simple hepatic steatosis to steatohepatitis, fibrosis, cirrhosis, and liver cancer. While the pathogenesis of NAFLD remains incompletely understood, a multihit model has been proposed that accommodates causal factors from a variety of sources, including intestinal and adipose proinflammatory stimuli acting on the liver simultaneously. Prior cellular and molecular studies of patient and animal models have characterized several common pathogenic mechanisms of NAFLD, including proinflammation cytokines, lipotoxicity, oxidative stress, and endoplasmic reticulum stress. In recent years, gut microbiota has gained much attention, and dysbiosis is recognized as a crucial factor in NAFLD. Moreover, several genetic variants have been identified through genome-wide association studies, particularly rs738409 (Ile748Met) in* PNPLA3* and rs58542926 (Glu167Lys) in* TM6SF2*, which are critical risk alleles of the disease. Although a high-fat diet and inactive lifestyles are typical risk factors for NAFLD, the interplay between diet, gut microbiota, and genetic background is believed to be more important in the development and progression of NAFLD. This review summarizes the common pathogenic mechanisms, the gut microbiota relevant mechanisms, and the major genetic variants leading to NAFLD and its progression.

## 1. Introduction

Nonalcoholic fatty liver disease (NAFLD) is the most common chronic liver disease in the world. It is present in 30% of the general adult population and found predominantly in obese people with high-fat diets and inactive lifestyles. In reality, NAFLD comprises a spectrum of hepatic abnormalities that are observable in liver histological slides, from a simple intrahepatic accumulation of fat (steatosis or nonalcoholic fatty liver, NAFL) to various degrees of necrotic inflammation (nonalcoholic steatohepatitis, NASH) [1–3]. Simple steatosis (i.e., NAFL) rarely progresses to advanced disease whereas, in approximately 20% of patients with NASH, it progresses to fibrosis and cirrhosis and potentially to hepatocellular carcinoma over a 15-year time period [4, 5]. The majority of patients with NAFLD are obese or even morbidly obese and have accompanying insulin resistance that plays a central role in the metabolic syndrome [6–9]. Thus, NAFLD is also deemed to be hepatic manifestation of metabolic syndrome which is a cluster of complex conditions including central obesity, hypertension, hyperglycaemia, hypertriglyceridemia, and low HDL (high density lipoprotein) that are predictive risk factors of cardiovascular disease, stroke, and diabetes [10, 11].

**Figure 1:**
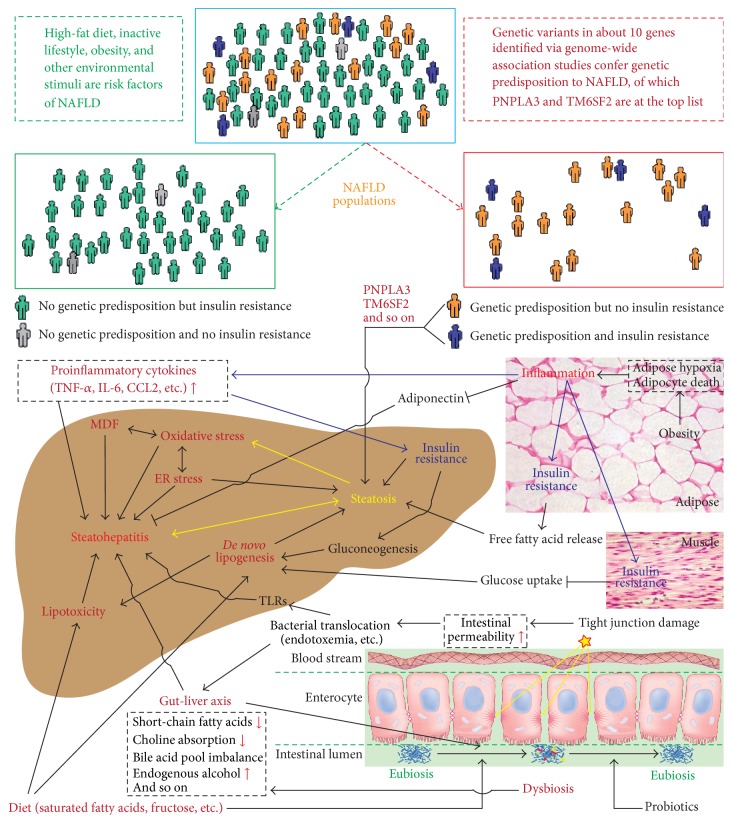
Overview at the pathogenesis of nonalcoholic fatty liver disease (NAFLD). The interplay between diet, microbiota, and host genetic variants plays a crucial role in the complex pathogenesis of NAFLD through a variety of mechanisms. The NAFLD patients can now be categorized into different populations based on their insulin sensitivity and genetic predisposition. Insulin resistance is at the center of the NAFLD pathogenic process, and a number of key factors are involved in the development of NAFLD, such as diet, dysbiosis, gut-liver axis, genetic predisposition genes (PNPLA3 and TM6SF2), oxidative stress, MDF (mitochondrial dysfunction), endoplasmic reticulum (ER) stress,* de novo* lipogenesis, lipotoxicity, and proinflammatory cytokines.

NAFLD has been considered a condition with a “two-hit” process of pathogenesis since 1998 when Day and James first proposed this hypothesis [12] with evidence from the Berson et al. study describing the role of lipid peroxidation in the liver injury [13]. Essentially, the first hit is the development of hepatic steatosis via accumulation of triglycerides in hepatocytes, which increases the vulnerability of the liver to various possible “second hits” that in turn lead to the inflammation, fibrosis, and cellular death characteristics of NASH. The second hit can be a variety of factors, such as oxidative stress, endoplasmic reticulum stress, proinflammatory cytokines, and gut-derived bacterial endotoxin. As it has evidently emerged that (1) accumulation of triglycerides in hepatocytes may be a protective mechanism from liver damage and (2) hepatic inflammation can precede the simple hepatic steatosis and can also be a cause of steatosis, it has been believed that many “hit” factors may act simultaneously leading to the development of NAFLD, which supports the multihit model proposed in 2010 [14]. Indeed, among the proposed hit factors, many can interact with each other, forming a vicious circle. Recent advances in metagenomics complicate the understanding of the pathogenesis of NAFLD further in that dysbiosis and host-microbiota interactions are now also implicated. Moreover, genome-wide association studies have discovered several promising candidate genes, serving as the genetic background for the disease. These genetic players appear to distinguish subgroups of NFLD patients from obese and insulin resistance associated populations. Although a high-fat diet and inactive lifestyles are typical risk factors for NAFLD, the interplay between diet, gut microbiota, and genetic background can play a crucial role in the development and progression of NAFLD. This review summarizes the common pathogenic mechanisms, the gut microbiota relevant mechanisms, and the major genetic variants leading to NAFLD and its progression (Figure 1).

## 2. Common Pathogenic Mechanisms of NAFLD

Hepatic steatosis is a prerequisite to making a histological diagnosis of NAFLD [2]. Several mechanisms may lead to steatosis, including (1) increased fat supply such as high-fat diet and excess adipose lipolysis; (2) decreased fat export in the form of very low density lipoprotein-triglyceride; (3) decreased free fatty *β*-oxidation; and (4) increased* de novo* lipogenesis (DNL) [2]. Molecular mechanisms responsible for the accumulation of fat in the liver are not fully understood; however, certain cytokines derived from inflammation sites, particularly from extrahepatic adipose tissues, can trigger this process. In addition, the enhancement of hepatic DNL is deemed to be a unique feature in steatosis. More importantly, insulin resistance appears to be at center stage for the massive metabolic dysregulations of NAFLD that initiate and aggravate hepatic steatosis. At a certain point, the simple steatosis transforms to steatohepatitis in about 20–30% of NAFLD patients. This breakthrough-like process is mediated by the interplay of multiple hit factors. Pathological features of NASH include simple hepatic steatosis and, more characteristically, liver cell damage and accompanying inflammation and/or fibrosis. Currently, a number of common pathogenic mechanisms have been proposed and characterized for the transition from simple steatosis to NASH, such as lipotoxicity, oxidative stress, mitochondrial dysfunction, and endoplasmic reticulum stress.

### 2.1. Adipose Tissue Inflammation

What exactly initiates adipose tissue inflammation in obesity is uncertain; but hypoxia and death of rapidly expanding adipocytes are believed to play a role [15]. Adipocytes under inflammation secrete cytokines and chemokines, particularly tumor necrosis factor-*α* (TNF-*α*), interleukin-6 (IL-6), and CC-chemokine ligand-2 (CCL2) [15, 16]. TNF-*α* was the first proinflammatory cytokine detected in adipose tissue and is involved in the regulation of insulin resistance. Studies indicated that neutralization of TNF-*α* activity by an anti-TNF-*α* monoclonal antibody improves insulin resistance and fatty liver disease in animals [17]. IL-6 is derived from many cells throughout the body including adipocytes. Serum levels of these cytokines correlate remarkably well with the presence of insulin resistance, and adipose tissue-derived TNF-*α* and IL-6 have been shown to regulate hepatic insulin resistance via upregulation of SOCS3, a suppressor of cytokine signaling [17]. CCL2 recruits macrophages to the adipose tissue, resulting in even more local cytokine production and perpetuating the inflammatory cycle; TNF-*α* and IL-6 induce a state of insulin resistance in adipocytes, which stimulates triglyceride lipolysis and fatty acid release into the circulation. At the same time, extrahepatic adipocytes are compromised in their natural ability to secrete adiponectin, an anti-inflammatory adipokine that facilitates the normal partitioning of lipid to adipocytes for storage [18]. Circulating adiponectin regulates hepatic fatty *β*-oxidation through AMP-activated protein kinase (AMPK) and acetyl-CoA carboxylase (ACC) signaling [19]. Together, these abnormalities accentuate fat loss from adipocytes and promote ectopic fat accumulation.

### 2.2. *De Novo* Lipogenesis (DNL)

Presumably lipogenesis in liver could be increased due to the steatotic nature of NAFLD. A number of prior studies have shown that diets enriched in both saturated fat and simple sugar carry a high risk of hepatic steatosis, at least in part, through enhanced DNL [20–23]. The role of DNL in the development of hepatic steatosis is further supported by a recent study in subjects with metabolic syndrome and a high content of liver fat [24]. A 3-fold higher rate of* de novo* fatty acid synthesis is seen in these subjects. In addition, specific dietary compositions may have different effects. Basically since carbohydrates are substrates for DNL, the amount of carbohydrate in the diet will positively influence the amount of DNL in the liver. Simple sugars are converted to fatty acids more readily than complex carbohydrates [25, 26], and fructose is a more potent inducer of DNL than glucose [27, 28]. This is also supported by epidemiologic evidence linking dietary fructose to hepatic steatosis and NASH [20, 29]. It is worth noting that dietary fat, particularly saturated fat, stimulates DNL by upregulating SREBP-1 (sterol responsive element binding protein), a key regulator of the lipogenic genes in the liver [30]. However, not all individuals with hepatic steatosis had increased DNL nor upregulated SREBP-1 expression, as observed in the Mancina et al. study showing a paradoxical dissociation between hepatic DNL and hepatic fat content due to the PNPLA3 148M allele [31].

### 2.3. Insulin Resistance

Studies have highlighted the fact that insulin resistance is a characteristic feature of NAFLD [7–9] and is caused by a variety of factors, including soluble mediators derived from immune cells and/or adipose tissue, such as TNF-*α* and IL-6 [32]. Serine phosphorylation of insulin receptor substrates by inflammatory signal transducers such as c-jun N-terminal protein kinase 1 (JNK1) or inhibitor of nuclear factor-*κ*B kinase-*β* (IKK-*β*) is considered one of the key aspects that disrupts insulin signaling [14]. On the other hand, insulin resistant subjects with NAFLD show reduced insulin sensitivity, not only at the level of the muscle, but also at the level of the liver and adipose tissue [7–9, 33], which can lead to a far more complex metabolic disturbance of lipid and glucose. However, not all people with NAFLD have increased insulin resistance, and NAFLD,* per se*, cannot be considered a cause for insulin resistance but rather a consequence as shown by studies in subjects genetically predisposed to NAFLD. Mutations in* PNPLA3* (patatin-like phospholipase domain containing 3) [34, 35],* TM6SF2* (transmembrane 6 superfamily member 2) [6, 36],* DGAT1* (diacylglycerol O-acyltransferase 1) [37], or hypobetalipoproteinemia [38, 39] genes are not related to increased insulin resistance except for severely obese individuals in which it is associated [40]. It is worth noting that insulin resistance is characterized not only by increased circulating insulin levels but also by increased hepatic gluconeogenesis, impaired glucose uptake by muscle, and increased release of free fatty acids and inflammatory cytokines from peripheral adipose tissues [41], which are the key factors promoting accumulation of liver fat and progression of hepatic steatosis (Figure 1).

### 2.4. Lipotoxicity

Studies have indicated that certain lipids can be harmful to hepatocytes in NAFLD. This is particularly true of the long-chain saturated fatty acids (SFAs) such as palmitate (C16:0) and stearate (C18:0), which are abundant in animal fat and dairy products and produced in the liver from dietary sugar. Under physiological conditions, SFAs are transported to mitochondria for *β*-oxidation or esterified for either excretion in the form of VLDL (very low density lipoproteins) or storage as lipid droplets. In the pathophysiology of NASH, multiple mechanisms are concurrently operative to produce liver injury in hepatocytes overwhelmed by SFA and by free cholesterol (FC) from* de novo* synthesis [42, 43]. FC accumulation leads to liver injury through the activation of intracellular signaling pathways in Kupffer cells (KCs), hepatic stellate cells (HSCs), and hepatocytes. The activation of KCs and HSCs promotes inflammation and fibrogenesis [44]. These lipids, including FC, SFA, and certain lipid intermediates from excessive SFA, can activate a variety of intracellular responses such as JNK1 and a mitochondrial death pathway, resulting in lipotoxic stress in the endoplasmic reticulum and mitochondria, respectively [42, 43, 45]. In addition, the toll-like receptor 4 (TLR4) is a pattern recognition receptor that activates a proinflammatory signaling pathway in response to excessive SFAs. This pathway is initiated by recruiting adaptor molecules such as toll/IL-1 receptor domain containing adaptor protein (TIRAP) and myeloid differentiation factor 88 (MyD88) that ultimately lead to activation of nuclear factor *κ*B with production of TNF-*α* [46].

### 2.5. Mitochondrial Dysfunction

Mitochondria are the most important energy suppliers of the cell and play a pivotal role in fatty acid metabolism. Fatty acid oxidation is able to be upregulated to compensate for some degree of increased deposition of fat; however, multiple studies have shown that liver ATP levels are decreased in NAFLD [47–49]. This discrepancy implicates mitochondrial dysfunction in the state of liver fat overload that is characteristic of NAFLD. Although the mechanisms responsible for the mitochondrial dysfunction remain poorly understood in NAFLD, reduced enzymatic activities of mitochondrial electron transport chain (ETC) complexes may be attributed to increased generation of reactive oxygen species (ROS) as a result of ETC leakage during mitochondrial *β*-oxidation in energy production (in the form of ATP) [50]. Studies have found that ROS can damage the ETC [51] and even cause mutations in the mitochondria DNA [52].

### 2.6. Oxidative Stress

In the context of increased supply of fatty acids to hepatocytes, oxidative stress can occur and be attributable to raised levels of reactive oxygen/nitrogen species (ROS/RNS) and lipid peroxidation that are generated during free fatty acid metabolism in microsomes, peroxisomes, and mitochondria [53–55]. Peroxidation of plasma and intracellular membranes may cause direct cell necrosis/apoptosis and megamitochondria, while ROS-induced expression of Fas-ligand on hepatocytes may induce fratricidal cell death. Recent studies support the idea that oxidative stress may be a primary cause of liver fat accumulation and subsequent liver injury, and ROS may play a part even in fibrosis development [56, 57]. Importantly, these species can initiate lipid peroxidation by targeting polyunsaturated fatty acids (PUFAs), resulting in the formation of highly reactive aldehyde products, such as 4-hydroxy-2-nonenal (4-HNE) and malondialdehyde (MDA). These reactive lipid derivatives have the potential to amplify intracellular damage by mediating the diffusion of ROS/RNS into the extracellular space, thus causing tissue damage.

### 2.7. Endoplasmic Reticulum (ER) Stress

The ER is a vast dynamic and tubular network responsible for the synthesis, folding/repair, and trafficking of a wide range of proteins [58]. Under pathological and/or stressful conditions such as NASH, it has been observed that ER efficiency in the protein-folding, repairing, and/or trafficking machinery is decreased while the demand of protein synthesis and folding/repair is increased [58, 59]. Such an imbalance between the load of needed protein-folding and the response-related capability of the ER is termed ER stress, which can lead to the accumulation of unfolded and/or misfolded proteins within the ER lumen. This type of cellular stress usually triggers an adaptive response, aimed at resolving ER stress, called unfolded protein response (UPR) [60–62]. The UPR is mediated by at least three different stress-sensing pathways: protein kinase RNA-like ER kinase (PERK), inositol-requiring protein 1*α* (IRE1*α*), and activating transcription factor 6 (ATF6) [62]. Coupled with inflammation, oxidative stress, insulin resistance, and apoptosis signaling, hepatic ER stress seems to play an important role in regulating the composition and size of lipid droplets as well as lipid synthesis, including cholesterol metabolism [58, 59, 63], through SREBP.

## 3. Microbiota Associated Mechanisms of NAFLD

Gut microbiota was first found to be altered in patients with chronic liver disease more than 80 years ago. Derangement of the gut flora, in particular small intestinal bacterial overgrowth (SIBO), occurs in a large percentage (20–75%) of patients with chronic liver disease. In recent years, the gut microbiome has gained much more attention due to the advancement of the high-throughput next-generation sequencing (NGS) technology. Prior studies of gut flora relied on culture dependent techniques, which were labor intensive and limited only to a countable number of species, as over 80% of the gut microbes are not cultivatable [64]. In contrast, NGS-based taxonomic assignments of the uncultured, undefined microbes into operational taxonomic units (OTUs) represent an effective and revolutionary approach for studies on highly complex gut microbiota, which is based on clustering of the 16S rRNA sequences derived from the NGS platforms. This approach allows the characterization of both composition and diversity of the intestinal microbiota. According to evidence from relevant studies, the gut microbiota may contribute to the pathogenesis of NAFLD through several mechanisms, including (1) increased production and absorption of gut short-chain fatty acids; (2) altered dietary choline metabolism by the microbiota; (3) altered bile acid pools by the microbiota; (4) increased delivery of microbiota-derived ethanol to liver; (5) gut permeability alterations and release of endotoxin; and (6) interaction between specific diet and microbiota. Most recently, Musso et al. brought up a new mechanism by that chronic kidney disease may mutually aggravate NAFLD and associated metabolic disturbances through multiple paths including altered intestinal barrier function and microbiota composition [65]. In fact, diet can affect the composition and diversity of gut microbiota; thus any changes of gut microbiota that are observed in a diet-stratified study should be interpreted with caution because these changes could be either a direct effect of specific diets or an indirect effect of the gut-liver interactive axis which has been proposed and observed recently [66, 67].

### 3.1. Short-Chain Fatty Acids (SCFAs) Relevant Mechanisms

In the intestine, SCFAs are produced in the distal small intestine and colon where nondigestible carbohydrates like resistant starch, dietary fiber, and other low-digestible polysaccharides are fermented by saccharolytic bacteria which include the phyla Bacteroidetes, Firmicutes, and Actinobacteria. Acetate and propionate are the main products of Bacteroidetes phylum and butyrate is mainly produced by Firmicutes phylum. As an energy precursor, SCFAs are implicated in the pathogenesis of NAFLD because of their possible contribution to obesity. The first evidence regarding SCFAs was from Turnbaugh et al. study [68] showing that the cecum of ob/ob mice has an increased concentration of SCFAs and that transplantation of germ-free mice with the gut microbiome from ob/ob mice caused greater fat gain than transplants from lean animals. In humans, increased production of SCFAs by the gut microbiota was also observed in overweight and obese people, compared to lean subjects [69]. In metagenomics analysis, the majority of studies showed that ob/ob mice [70] and obese patients [71] exhibit reduced abundance of Bacteroidetes and proportionally increased abundance of Firmicutes. However, how these ratio changes affect energy imbalance leading to obesity and its complications including NAFLD needs further functional and species-level analyses. In fact, SCFAs have more beneficial effects than their obesity-causing effects in general [72]. Beneficial effects of SCFAs are through several ways, such as immunoregulation, enhanced intestinal barrier function, acting as a histone deacetylase 1 (HDAC) inhibitor to decrease expression of lipogenic genes and to increase carnitine palmitoyltransferase 1A expression [72], and a peroxisome proliferator-activated receptor-*γ*- (PPAR*γ*-) dependent mechanism, shifting metabolism in adipose and liver tissue from lipogenesis to fatty acid oxidation [73].

### 3.2. Dietary Choline Mechanism

Dietary choline is required for very low density lipoprotein synthesis and hepatic lipid export; and dietary choline-deficiency has been linked with a variety of conditions including hepatic steatosis. Buchman et al. [74] found that, in patients with parenteral nutrition, diets deficient in choline can lead to increased hepatic steatosis, which can be reversed with choline supplementation. This study suggests a role of choline in fat export out of the hepatocytes. Recent studies indicate a role of the intestinal microbiota in the conversion of dietary choline to toxic methylamine, a substance that not only mimics a choline-deficient diet by decreasing effective choline levels but also exposes the host to an inflammatory toxic metabolite [75]. Very recently, a metagenomic analysis of the microbial communities living in the intestinal tracts of 15 women with a choline-depleted diet revealed that increased Gammaproteobacteria abundance and decreased Erysipelotrichi abundance were protective against developing steatosis [76].

### 3.3. Bile Acid Pool Related Mechanisms

Within hepatocytes, bile acids are synthesized from cholesterol through enzymatic pathways and then conjugated with either glycine or taurine before secretion into bile and released into the small intestine. In the small intestine, conjugated bile acids not only assist in lipid absorption and transport but have also been increasingly recognized to function as nuclear receptor binders and to have a putative role in altering the microbiome [77]. On the other hand, bacteria within the intestine can also chemically modify bile acids and thereby alter the composition of the bile acid pool [78, 79]. Besides the classic role as detergents to facilitate fat absorption, bile acids have also been recognized as important cell signaling molecules regulating lipid metabolism, carbohydrate metabolism, and inflammatory response [80]. These molecular functions are mediated through their binding and activation of the nuclear hormone receptor, farnesoid X receptor (FXR), and the G protein coupled cell surface receptor TGR5 [81]. Intestinal FXR activity upregulates endocrine FGF19 expression, which inhibits hepatic bile acid synthesis via CYP7A1 signaling [82]. McMahan et al. showed that activation of bile acid receptors with a receptor agonist was able to improve NAFLD histology in an obese mouse model [83]. Due to the nature of the complex interplay between the microbiome and the host bile acid pool, further studies are required in the context of risk for NAFLD and NASH.

### 3.4. Endogenous Alcohol Theory

The possible role for endogenous alcohol in NAFLD was first implicated in ob/ob mice. Cope et al. found that alcohol in the breath of obese animals is higher than that of lean animals [84], but they could not find any difference in the breath alcohol concentration between NASH patients and lean controls in a human study [85]. Recently, Zhu et al. found that NASH patients exhibited significantly elevated blood ethanol levels, while similar blood ethanol concentrations were observed between healthy subjects and obese non-NASH patients [86]. Further, in this metagenomics study, the composition of NASH microbiomes was found to be distinct from those of healthy and obese microbiomes, and* Escherichia* stood out as the only abundant genus that differed between NASH and obese patients. Because* Escherichia* are ethanol producers, this finding is in agreement with their previous report that alcohol-metabolizing enzymes are upregulated in NASH livers [87]. However, Engstler et al. provided evidence against the alcohol theory [88]. In their study, ethanol levels were similar in portal vein and chyme obtained from different parts of the GI tract between groups, while ethanol levels in vena cava plasma were significantly higher in ob/ob mice, suggesting that more ethanol was not metabolized in the liver due to a significantly lower ADH activity observed in these ob/ob mice. They proposed that increased blood ethanol levels in patients with NAFLD may result from insulin-dependent impairments of ADH activity in liver tissue, rather than from an increased endogenous ethanol synthesis. Thus, the alcohol theory currently faces conflicting results from different investigators. To clarify these conflicting results, de Medeiros and de Lima have provided an interesting mechanistic framework explaining how NAFLD might be an endogenous alcohol fatty liver disease (EAFLD) [89]. However, this framework requires experimental evidence to be validated.

### 3.5. Intestinal Permeability and Endotoxemia

The gut microbiota plays a part in maintaining the integrity of the intestinal barrier [90]; and changes in the composition of microbiota can lead to increased intestinal permeability and subsequent overflow of harmful bacterial by-products to the liver that in turn triggers hepatic inflammation and metabolic disorders. Endotoxin, that is, lipopolysaccharide (LPS), is derived from Gram-negative bacteria, and it has long been implicated in chronic liver diseases. The first evidence in support of a role for LPS in the pathogenesis of NASH was the observation that endotoxemia readily induces steatohepatitis in obese rats and mice [91]. Further, murine NAFLD models of bacterial overgrowth develop compositional changes of the gut microbiota and present increased intestinal permeability, with a concurrent reduction in the expression of tight junction proteins [92]. In human studies, Miele et al. found evidence of a disruption in the intestinal barrier of biopsy-proven NAFLD patients, along with an increased rate of small bowel bacteria overgrowth, suggesting that alterations in the microbiome may have contributed to disruption of gut barrier integrity [93]. In addition, high-fat diets may facilitate LPS uptake through elevated chylomicron production in intestinal epithelial cells [94]. On the other hand, Yuan et al. did not find the correlation between Gram-negative bacteria abundance and the concentration of serum endotoxin and there was no endotoxemia in the majority of pediatric NASH patients [95], highlighting the multihit hypothesis for the pathogenesis of NASH. Nonetheless, LPS and other exogenous stimuli are responded to first by innate immunity through pattern recognition receptors such as toll-like receptors (TLRs) and NOD-like receptors (NLRs). Although TLRs might respond to nutritional lipids such as free fatty acids [96], studies have implicated the importance of LPS-TLR4/TLR9 signaling in the pathogenesis of NAFLD. Both TLR4- and TLR9-deficient mice are protected from high-fat diet-induced inflammation and insulin resistance [97, 98], while mice deficient in TLR5 develop all features of metabolic syndrome including hyperphagia, obesity, insulin resistance, pancreatic inflammation, and hepatic steatosis [99]. Metagenomic analysis indicated that TLR5 deficiency affected the composition of the gut microbiota and, remarkably, transfer of the microbiota from TLR5^−/−^ mice to healthy mice resulted in transfer of disease [99]. Moreover, Wlodarska et al. found that NOD-like receptor family pyrin domain containing 6 (NLRP6) inflammasome deficiency leads to an altered transmissible, colitogenic gut microbiota [100]. When fed with a methionine and choline-deficient diet (MCDD), these inflammasome deficient mice developed NASH with significantly higher severity than wild-type animals [101].

### 3.6. Saturated Fatty Acids

It has been well known that animal meats are rich in saturated fatty acids (SFAs) which are highly correlated to an increased risk of obesity, diabetes, and cardiovascular diseases. Many studies have indicated that saturated fatty acids are more toxic than their unsaturated counterparts [102, 103]. It is worth noting that SFAs are protective in alcohol induced fatty liver disease [104–106]. However, in liver and hepatocytes not exposed to alcohol, SFAs appear to promote apoptosis and liver injury [107, 108]. It has been shown that SFAs increase the saturation of membrane phospholipids, thus initiating unfolded protein response (UPR) and leading to ER stress [108, 109]. SFAs also affect mitochondrial metabolism and promote ROS accumulation [23]. Furthermore, hepatocyte apoptosis has been shown to be dependent on the activation of JNK stress signaling pathways that respond to prolonged ER and oxidative stress [109]. In addition, SFAs can interact with gut microbiota to affect the progression of liver injury. For instance, by analyzing changes in the intestinal metagenome and metabolome of alcohol-fed mice, Chen et al. recently found that synthesis of saturated long-chain fatty acid is significantly reduced when compared with normal-chew mice and that supplementation of saturated long-chain fatty acids recovers intestinal eubiosis and reduces ethanol-induced liver injury in mice [110]. Moreover, de Wit et al. observed an overflow of SFAs to the distal intestine in mice on a high-SFA diet, which, rather than obesity itself, reduced microbial diversity and increased the Firmicutes-to-Bacteroidetes ratio in the intestine. Such a typical obesity microbiota profile stimulated by SFAs favors the development of obesity and hepatic steatosis [103].

### 3.7. Fructose

Fructose has been utilized as artificial sweetener in many commercial soft drinks that are consumed largely by adolescents and in a variety of social circumstances. A number of studies have found that excess fructose consumption is involved in the pathogenesis of NAFLD and that upregulated* de novo* lipogenesis and inhibited fatty acid *β*-oxidation are distinct metabolic processes for the development of hepatic steatosis in individuals with NAFLD [20, 24, 111–113]. Further, Abdelmalek et al. observed that increased fructose consumption is associated with a higher fibrosis stage in patients with NAFLD, independent of age, sex, BMI, and total calorie intake [29]. Using a fructose-induced NAFLD mouse model, recent studies with metagenomics analysis found that fructose significantly decreased* Bifidobacterium* and* Lactobacillus* and tended to increase endotoxemia [114, 115]. Several probiotic bacterial strains of* Lactobacillus* protect mice from the development of high-fructose-induced NAFLD [116–118]. In addition, increased expression of TLRs has been implicated in the development of fructose-induced hepatic steatosis [119].

## 4. Genetic Background of NAFLD

Genomic variations that have a causative effect on the development of human diseases can be divided into two groups: ones in rare diseases and ones in common diseases. The former follow Mendelian inheritance patterns that are characterized by a single, highly penetrant but uncommon mutation in a specific gene being necessary and sufficient to cause the disease. The latter consist of causative mutations that are not subject to negative selection pressures, and disease susceptibility is due to the combined effects of multiple relatively common causative polymorphisms (minor allele frequency 1–5%) that are carried by affected individuals. Like most common diseases, NAFLD has been implicated in an inherited component to susceptibility, meaning that genetic variation does influence disease risk. As reviewed by Macaluso et al. in 2015 [120], dozens of genes with multiple polymorphisms have been discovered in genome-wide association studies (GWAS) that may be responsible for risk of NAFLD in certain populations. It is believed that as more large scale GWAS are complete, more genes could be identified. For instance, in early 2016 while we were preparing this review, a novel variant MBOAT7 rs641738 was reported to be associated with the development and severity of NAFLD in individuals of European descent [121]. Among all reported genes, only two of them (*PNPLA3* and* TM6SF2*) have been identified as potential genetic modifiers in more than one large scale study [120, 122], which are the focus of our review. According to genotypes in those key genes and sensitivity to insulin, NAFLD patients can be categorized into different subpopulations (Figure 1).

### 4.1. *PNPLA3* (Patatin-Like Phospholipase Domain Containing 3)

The* PNPLA3* gene (adiponutrin) encodes a transmembrane polypeptide chain exhibiting triglyceride hydrolase activity [123], which is highly expressed on the endoplasmic reticulum and lipid membranes of hepatocytes and adipose tissue [124]. It is also reported that* PNPLA3* is highly expressed in human stellate cells. The encoded protein has retinyl esterase activity and allows retinol secretion from hepatic stellate cells while the mutation causes intracellular retention of this compound [125–127]. As the first genome-wide association study with strong evidence for NAFLD, a report from Romeo et al. in 2008 showed that a genetic variant, an allele in* PNPLA3* (rs738409[G], encoding Ile148Met), confers susceptibility to the disease in individuals of several western populations [128]. This genetic variant was associated with increased liver fat and hepatic inflammation and fibrosis. This finding has subsequently been reproduced with solid evidence as shown in a meta-analysis comprising 16 studies [129]. Compared with noncarriers, homozygous carriers of the variant had a 73% higher liver fat content, a 3.2-fold greater risk of high necroinflammatory scores, and a 3.2-fold greater risk of developing fibrosis. The association between the* PNPLA3* variant and steatosis or severity of histological liver disease has been widely observed in the majority of subsequent genome-wide association studies [130] and several case-control studies, including those in Chinese, Korean, and Japanese populations [131–133]. It is worth noting that the link between* PNPLA3* I148M variant and NAFLD is independent of metabolic syndrome (MS) and its features; that is, most of patients carrying this variant are not associated with obesity, diabetes, and atherogenic dyslipidemia, as demonstrated in the recent meta-analysis [129]. Furthermore, the* PNPLA3* genotype seems to also influence steatosis development in patients with hepatitis B and hepatitis C and alcohol abuse, and it has been independently associated with the progression of hepatitis, including fibrosis, cirrhosis, and HCC occurrence [134–136]. The association between the* PNPLA3* variant I148M and the risk of HCC development has been robustly validated in patients with NAFLD [137, 138], and it has been estimated that the homozygous carriers of the p.148M mutation carry a 12-fold increased HCC risk as compared to p.I148 homozygotes [139]. Finally, as described earlier, subpopulations of NAFLD patients with PNLA3 mutation are not associated with insulin resistance, a hallmark of metabolic syndrome. Collectively, it seems that a distinct entity might exist in which the* PNPLA3* risk allele appears to be a major driver of disease progression in combination with viral infection, alcohol abuse, lifestyle (unhealthy diet and inactivity), and/or nonlifestyle (cryptogenic) causes, for example,* PNPLA3*-associated steatohepatitis (“PASH”) [140].

### 4.2. *TM6SF2* (Transmembrane 6 Superfamily Member 2)

Another widely validated and intriguing genetic player in NAFLD is the nonsynonymous variant rs58542926 (c.449 C>T) within the* TM6SF2* gene at the 19p13.11 locus, which encodes an E167K amino acid substitution. The role of variant E167K in* TM6SF2* was first described by Kozlitina et al. [36] in an exome-wide association study in a multiethnic, population-based cohort, highlighting the association of the* TM6SF2* variant with higher serum alanine aminotransferase (ALT) and aspartate aminotransferase (AST) levels—as surrogates for NASH—and with reduced plasma levels of triglycerides and low density lipoprotein- (LDL-) cholesterol. In addition, they performed a functional analysis for* TM6SF2* in mouse models by silencing the gene via adeno-associated viral vectors. Silencing of the gene showed a 3-fold increase in hepatic triglycerides levels and a decrease in plasma levels of triglycerides, LDL- and high density lipoprotein- (HDL-) cholesterols, and very low density lipoprotein (VLDL). Overall, their results demonstrated that the* TM6SF2* gene regulated hepatic triglyceride secretion and that the functional impairment of* TM6SF2* promoted NAFLD. An association between the* TM6SF2* rs58542926 SNP and the severity of liver disease has also been found in patients with biopsy-proven NAFLD in a recent study reported by Liu et al. [141]. More intriguingly, the E167K variant in* TM6SF2* seems able to disconnect the risk of NAFLD/NASH progression from cardiovascular risk, which is supported mainly by the Dongiovanni et al. study [142] showing that 188 (13%) out of 1201 subjects who underwent liver biopsy for suspected NASH were carriers of the E167K variant and that these carriers had lower serum lipid levels than noncarriers, more severe steatosis, necroinflammation, ballooning, and fibrosis and were more likely to have NASH and advanced fibrosis after adjusting for metabolic factors and the* PNPLA3* I148M risk variant. In addition, E167K carriers had lower risk of developing carotid plaque; and in Swedish obese subjects assessed for cardiovascular outcomes, E167K carriers had higher ALT and lower lipid levels but also a lower incidence of cardiovascular events. Consequently, carriers of the* TM6SF2* E167K variant seem to be more at risk for progressive NASH, but at the same time they could be protected against cardiovascular diseases [143].

## 5. Interplay between Diet, Microbiota, and Host Genetics

One of the biggest lessons we learned from the metagenomic studies so far is that constitutive profiles of gut microbiota can determine liver pathology in response to a high-fat diet (HFD) in mice, reflecting a kind of interactive effect between diet and gut microbiota, that is, a net effect after the interplay. For instance, in a transplantation experiment [144], Le Roy et al. selected donor mice at first, based on their responses to a HFD. The “responders” developed hyperglycaemia and had a high plasma concentration of proinflammatory cytokines, and the “nonresponders” were normoglycaemic and had a lower level of systemic inflammation, although both developed comparable obesity on the HFD. Germ-free mice were then colonized with intestinal microbiota from either the responder or the nonresponder mice and then fed the same HFD. The responder-receiver (RR) group developed fasting hyperglycaemia and insulinaemia, whereas the nonresponder-receiver (NRR) group remained normoglycaemic. In contrast to NRR mice, RR mice developed hepatic macrovesicular steatosis, which was confirmed by a higher liver concentration of triglycerides and increased expression of genes involved in* de novo* lipogenesis. Pyrosequencing of the 16S ribosomal RNA genes revealed that RR and NRR mice had distinct gut microbiota including differences at the phylum, genera, and species levels. These results suggest that the gut microbiota can contribute to the development of NAFLD, independent of obesity but acting like a constitutional background of a host organ system. The interrelationship between diet, gut microbiota, and host genetics has been unraveled further in a recent study reported by Ussar and coworkers [145]. In this study, they utilized three commonly used inbred strains of mice: obesity/diabetes-prone C57Bl/6J, obesity/diabetes-resistant 129S1/SvImJ, and obesity-prone but diabetes-resistant 129S6/SvEvTac mice. Analysis of metabolic parameters and gut microbiota in all strains and their environmentally normalized derivatives revealed strong interactions between microbiota, diet, breeding site, and metabolic phenotype. More intriguingly, environmental reprogramming of microbiota resulted in obesity-prone 129S6/SvEvTac mice becoming obesity resistant. This study suggests that development of obesity/metabolic syndrome is the result of interactions between gut microbiota, host genetics, and diet.

## 6. Conclusions

NAFLD is best considered a multietiology disease trait, meaning that it is not caused by a single gene mutation genetically and is not associated with only a single factor environmentally; but it is the outcome of genetic variant-environmental factor interplay determining disease phenotype and progression. The genetic variants in* PNPLA3* and* TM6SF2* are only responsible for ~50% of NAFLD patients [120], and majority of* PNPLA3*-associated NAFLD patients are not obese and have no insulin resistance and its related diabetes and cardiovascular diseases [140]. In fact, like many common diseases, NAFLD is polygenic, where the heritable component to susceptibility variously accounts for up to 30–50% of relative risk [130]. Moreover, individual environmental factors, particularly the specific diets, interact with gut microbiota up front before a final beneficial or damaging signal is sent. Whether environmental factors, including lifestyle, are the cause of NAFLD will be steered by the interaction with the host genetics as well as the constitutional profile of gut microbiota. Thus, careful, multifaceted study designs are warranted in future analysis in order to “catch” the true causes to NAFLD.
